# Pharmacophore Modeling and Docking Studies on Some Nonpeptide-Based Caspase-3 Inhibitors

**DOI:** 10.1155/2013/306081

**Published:** 2013-09-08

**Authors:** Simant Sharma, Arijit Basu, R. K. Agrawal

**Affiliations:** ^1^Pharmaceutical Chemistry Research Laboratory, Department of Pharmaceutical Sciences, Dr. Harisingh Gour University, Sagar, Madhya Pradesh 470003, India; ^2^Department of Pharmaceutical Sciences, Birla Institute of Technology, Mesra, Ranchi, Jharkhand 835 215, India

## Abstract

Neurodegenerative disorders are major consequences of excessive apoptosis caused by a proteolytic enzyme known as caspase-3. Therefore, caspase-3 inhibition has become a validated therapeutic approach for neurodegenerative disorders. We performed pharmacophore modeling on some synthetic derivatives of caspase-3 inhibitors (pyrrolo[3,4-c]quinoline-1,3-diones) using PHASE 3.0. This resulted in the common pharmacophore hypothesis AAHRR.6 which might be responsible for the biological activity: two aromatic rings (R) mainly in the quinoline nucleus, one hydrophobic (H) group (CH_3_), and two acceptor (A) groups (–C=O). After identifying a valid hypothesis, we also developed an atom-based 3D-QSAR model applying the PLS algorithm. The developed model was statistically robust (*q* ^2^ = 0.53; pred_*r* ^2^ = 0.80). Additionally, we have performed molecular docking studies, cross-validated our results, and gained a deeper insight into its molecular recognition process. Our developed model may serve as a query tool for future virtual screening and drug designing for this particular target.

## 1. Introduction

Neurodegenerative disorders like Alzheimer's disease (AD) and Huntington's disease [1] are major consequences of abnormal apoptosis of neurons. This abnormal apoptosis is also responsible for occurrence of myocardial infarction [2] and liver diseases [3]. Recently available drug treatments are partially effective, as they only improve the function of the neurons that are still alive, but they do not influence the underlying mechanisms leading to their death [4].

Caspases, a group of cysteine proteases, are proteolytic in nature and key executioners of apoptosis [5]. Caspase-3 is of particular interest among these caspases as it is responsible for the progression of AD. Caspases can be classified into two broad categories: firstly, initiator caspases (caspase-2, caspase-8, caspase-9, and caspase-10), and secondly, effector caspases (caspase-3, caspase-6, and caspase-7). Initiator caspases generally act in the early stages of a proteolytic cascade, whereas effector caspases act downstream and are involved in the cleavage of specific cellular proteins [6].

The initiation of caspase cascade reaction can only be regulated by caspase inhibitors. Inhibitors of caspase-3 were described as promising cardioprotectants [7], neuroprotectants [8], and hepatoprotectants [9]. Many of compounds such as isatins [10], peptidealdehydes [11], and homophthalimides [12] have been reported to be caspase inhibitors because of having electron-deficient carbonyls that interact with the target site and show inhibitory responses. Various studies are available on pharmacophore- and structure-based drug designing of peptide and nonpeptide caspase-3 inhibitors including different caspase-3 PDB IDs. Binding interactions of these inhibitors are almost similar in binding pocket of caspase-3. Lakshmi et al. have performed ligand-and structure-based virtual screening on caspase-3 inhibitors. They used a different nucleus, but the same activity assay. They have used PDB ID 1PAU and reported almost similar interactions (hydrogen-bond interactions between the compound and Arg 207 and between Ser 209 and Trp214) as we are reporting in the present study [13]. All of the above studies are available on different nuclei and different caspase-3 PDB IDs, but till now no study is available on pyrrolo[3,4-c]quinoline-1,3-diones using PDB ID: 1GFW. Generally, potent caspase-3 inhibitors reported till date are peptides in nature [14–17]. However, such inhibitors often acquire poor cell permeability and low metabolic stability [18]. This problem can be conquered by developing nonpeptide small-molecule inhibitors of caspase-3. 

Pharmacophore modeling has been one of the important and successful ligand-based approaches for new drug discovery in the last few years [19–21]. It has been defined as a term which depicts the configurations of chemical features that are common to all of the ligands. A pharmacophore hypothesis collects the common features distributed in the three-dimensional space representing groups in a molecule that participate in important interactions between the drug and the active site [22]. In the search for a new drug, a pharmacophore often serves as a template for the desired ligand [23, 24].

In the present study, we have developed an atom-based 3D pharmacophore model using PHASE module, which provides the key structural features required for the biological activity. Furthermore, binding interactions of active molecules were analyzed in binding pocket of caspase-3 by using Glide the in SP mode. Atom-based 3D-QSAR model generates cubes which emphasize the structural features required for caspase-3 inhibition. This piece of information can be useful for further designing of more potent caspase-3 inhibitors.

## 2. Material and Methods

The computational work was performed on Red Hat Linux Enterprise 3.0 with Intel Pentium Core 2-Duo Processor, 1 GB RAM and 120 GB (hard disk). All of the structures were built on Maestro 8.5, a module of Schrodinger [25]. Furthermore, PHASE 3.0 module [26] was used for pharmacophore modeling.

A dataset of 82 compounds was selected from two series of caspase-3 inhibitors having similar basic nuclei and activity assays [27, 28].

The structures were built followed by clean geometry and energy minimization with OPLS_2005 force field method. The activity data for each compound were taken as negative logarithms of IC_50_ values (molar). All of the structures of compounds with their observed and predicted activities data are given in Tables 1 and 2.

### 2.1. Pharmacophore Modeling

PHASE, version 3.0, was used for pharmacophore elucidation and QSAR model building. It provides six built-in types of pharmacophore features: hydrogen-bond acceptor (A), hydrogen-bond donor (D), hydrophobe (H), negative ionizable (N), positive ionizable (P), and aromatic ring (R). Ligands were processed with the LigPrep program to assign protonation states appropriate for pH 7.0. All of the molecules were considered to be active for building the hypothesis. Conformer generation for each ligand was carried out with the ConfGen method where the maximum number of conformers was set as 100 per ligand, and total steps per rotatable bond were set as 100 (Table 3). For this method, sampling of conformational space was done in rapid mode. Maximum relative energy difference was 10 Kcal/mol. Distance-dependant dielectric salvation treatment was performed. Both pre- and post-process minimization steps were set as 100 and 50, respectively. The minimization and energy calculation were done using OPLS_2005 force field method. The default pharmacophore feature definitions were used for site generation. After site generation, common pharmacophore was searched from a set of variants which was generated by a systematic variation of the number of sites. Variant AAHRR for which All the compounds were matched was searched to generate the best common pharmacophore hypothesis (AAHRR.6) based on the survival score by PHASE. The minimum distance between any two sites was set to 2 Å, and the maximum tree depth was set to 4 with a final box size of 1 Å. The survival score of hypothesis can be determined by using the following formula: survival score = (Vector score) + (Site score) + (Volume score) + (Selectivity score) + (Number of actives that match the hypothesis − 1) − (Reference-ligand relative conformational energy) + (Reference-ligand activity). Hypothesis AAHRR.6 was selected as the best hypothesis because it has a higher survival score (3.785) than others (Table 4). The highest survival score for common pharmacophore hypothesis gives the best alignment of the active ligands to this hypothesis. This alignment gives the fitness to all of the inhibitors. The best aligned ligand gives the maximum fitness. The top-scored hypothesis was then used to build atom-based 3D-QSAR model. Pharmacophore-based QSARs do not consider ligand features beyond the pharmacophore model, such as possible steric clashes with the receptor. This requires consideration of the entire molecular structure; therefore, an atom-based QSAR model is more useful in explaining the structure-activity relationship. In atom-based QSAR, a molecule is treated as a set of overlapping van der Waals spheres [22].

Pharmacophore model development was performed after dividing the dataset of 82 compounds into training (62 compounds) and test sets (20 compounds) and after applying PLS factor 4 and grid spacing 1 Å. The training set selection was done on the basis of the information contained in terms of both structural features and biological activity ranges of molecules. The training set included the compounds that cover all ranges of activities (high, moderate, and low active) [29]. The training set of 62 compounds was used for atom-based 3D-QSAR model development. In this study, most active compounds (compounds 49 and 58) were superposed in binding pocket with respect to common pharmacophore hypotheses to explain why they are similar in activity. 

### 2.2. Model Validation

This is done to test the internal stability and the predictive ability of the QSAR models. Developed QSAR models were validated by the following procedures [30]. 

#### 2.2.1. Internal Validation

Internal validation was carried out using the leave-one-out (*q* ^2^, LOO) method. For calculating *q* ^2^, each molecule in the training set was eliminated once, and the activity of the eliminated molecule was predicted by using the model developed by the remaining molecules. The *q* ^2^ was calculated using the following equation which describes the internal stability of a model:

(1) $$\begin{array}{c} q^{2}=1-\frac{\sum_{i=1}^{N}{\left(y_{\text{pred},i}{-y}_{i}\right)}^{2}}{\sum_{i=1}^{N}{\left(y_{i}-y_{m}\right)}^{2}}, \end{array}$$

where *y*_*i*_ and *y*_pred,*i*_ are the actual and the predicted activities of the *i*th molecule in the training set, respectively, and *y*_*m*_ is the average activity of all of the molecules in the training set.

#### 2.2.2. External Validation

For external validation, the activity of each molecule in the test set was predicted using the model developed by the training set. The pred_*r* ^2^ value is calculated as follows:

(2) $$\begin{array}{c} {\text{pred}\_r}^{2}=1-\frac{\sum_{i=1}^{N}{\left(y_{\text{pred},i}{-y}_{i}\right)}^{2}}{\sum_{i=1}^{N}{\left(y_{i}-y_{m}\right)}^{2}}, \end{array}$$

where *y*_*i*_ and *y*_pred,*i*_ are the actual and the predicted activities of the *i*th molecule in the test set, respectively, and *y*_*m*_ is the average activity of all of the molecules in the training set.

### 2.3. Docking Studies

Docking study was performed on Glide 5.0 module of Schrodinger [31]. X-ray crystal structure of caspase-3 (PDB ID: 1GFW with resolution of 2.80 Å) was used for docking study. The selection of PDB ID was done on the basis of similarities of cocrystallized ligand [isatin sulfonamide; or (s)-1-methyl-5-(2-(phenoxymethyl) pyrrolidin-1-ylsulfonyl)indoline-2,3-dione] to the reported caspase-3 inhibitors. Before starting the docking, preparation of protein was performed by removal of cocrystallized ligand and coupled water molecules followed by addition of hydrogen atoms. Then, protein was minimized with force field OPLS_2005. This refined protein structure was used for receptor grid generation by using default parameters. Grid size is defined according to the size of the cocrystallized ligand. All amino acids within 10 Å of the cocrystallized ligand were included in the grid file generation. All of the minimized inhibitors were docked into the receptor, and the best pose of each inhibitor was observed. The purpose of docking in this study is only to show the interactions of the top two most active compounds (compounds 49 and 58) with active site residues required for biological response. 

## 3. Results and Discussion

### 3.1. Atom-Based 3D-QSAR Model Development

**Figure 1:**
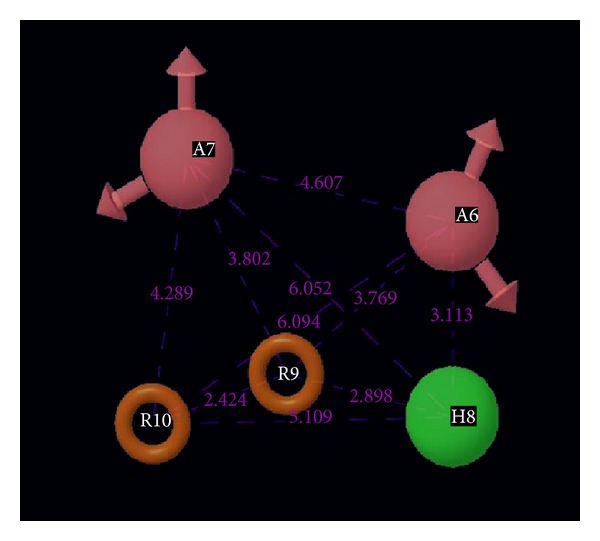
Common pharmacophore hypothesis and distances between pharmacophoric sites. Pink spheres with arrows show hydrogen-bond acceptor with lone pairs of electron. Green sphere shows hydrophobe, and yellow rings show ring aromatics.

We developed an atom-based 3D-QSAR model by using a grid spacing of 1.0 Å and a maximum PLS factor of four. The model was developed using five-point common pharmacophore hypothesis AAHRR.6 (Figure 1), which consists of two hydrogen-bond acceptors (A), one hydrophobe (H), and two aromatic rings (R). This hypothesis (AAHRR.6) was selected on the basis of the highest survival score, and these points were denoted as A6A7H8R9R10. The 3D-QSAR model developed by PLS methodology has admirable significant statistics (*R* ^2^ = 0.925, *F*value = 175.9, SD = 0.298, *Q* ^2^ = 0.798, *q* ^2^ = 0.53, and pred_*r* ^2^ = 0.80), which are given in Table 5.

**Figure 2:**
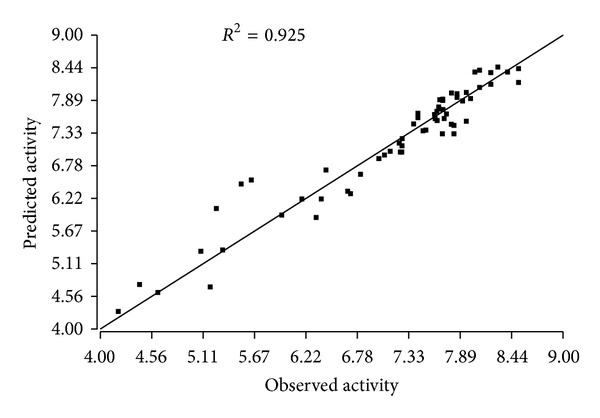
Plot between observed and predicted biological activities of the training set of compounds.

**Figure 3:**
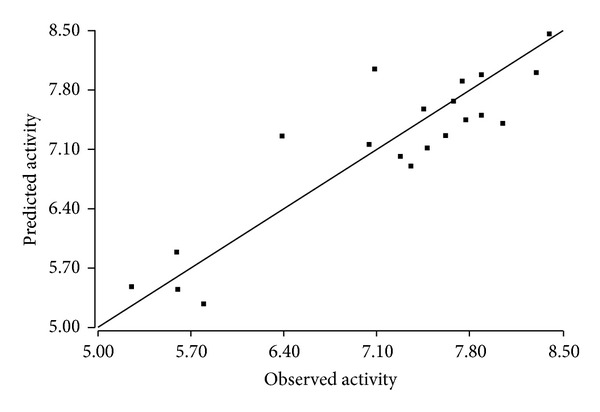
Plot between observed and predicted biological activities of the test set of compounds.

The plots between the observed and the predicted activities were made for both the training and test sets (Figures 2 and 3). The higher values of *R* ^2^ and *Q* ^2^ in the training and the test set, respectively are clearly indicated by the points lying extremely near to the best-fit line. On the basis of the statistical data, the model can be assumed to be statistically fit. A QSAR model is useful when it can predict an external dataset not used in model building, that is, for a test set. We obtained high pred_*r* ^2^ value (pred_*r* ^2^ = 0.80) suggesting a significant external predictive ability of the QSAR model. 

**Figure 4:**
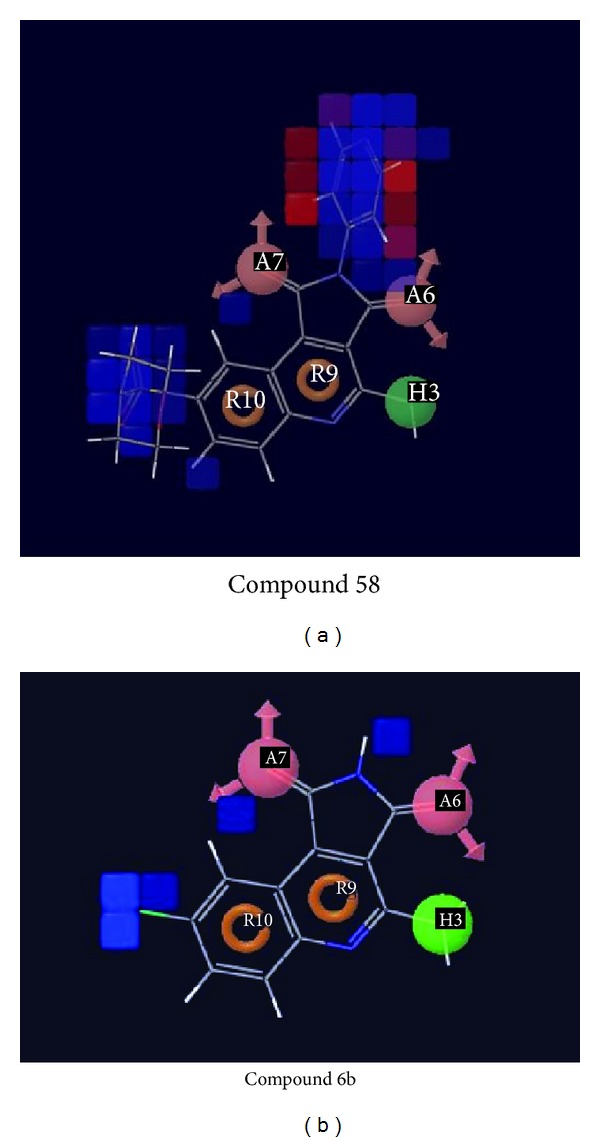
Pictorial representation of cubes for the most active (compound 58) and the least active (compound 6b) compounds where blue and red cubes show favorable and unfavorable regions for activity.

The developed 3D-QSAR model can be visualized as a cluster of cubes, which provided additional information about the structural features required for activity. The blue cubes point out favorable features, and the red cubes point out unfavorable features for activity. A comparative study of these favorable and unfavorable features for the most active (58) and the least active (6b) compounds is shown in Figure 4. In the most active compound (58), blue cubes around different positions of the basic nucleus can be explained as follows: blue cubes around position 8 explain why bulky groups with electron-withdrawing capacity are favorable for activity, around position 2, they explain why heteroaryl substitution is favorable for activity, and around the A7 position, they explain that the electronegative atom directly attached to C_1_ carbon is the favorable feature for activity. Less active compounds (compounds 6b, 6c, 6e, and 6i) have only electron-withdrawing substituents, but they lack any bulky substitution at position 8. This observation explains that bulky group substitution with electron-withdrawing capacity around position 8 is crucial for activity. Our observations are consistent with those of the earlier reports [27, 28] which reported that caspase-3 inhibitory activity is extremely dependent on the nature of substituents at positions 2 and 8. We observed that electron-withdrawing group at position 8, and heteroaryl group at position 2 are intrinsic for activity [27, 28]. 

In our previous study [32] on caspase-3 inhibitors, we reported a 2D-QSAR model. The earlier developed model suggested that incorporating the functional groups that have less LUMO energies, that is, more electron affinities, is favorable for biological activity. In our present study, we can also work out similar patterns, as given in Figure 4. Our currently developed 3D-QSAR model agrees to our earlier developed 2D-QSAR model. Additionally, through the developed 3D-QSAR, we got insights that are more mechanistic. 

We have successfully developed a QSAR model that is statistically robust and consistent with earlier observations. It also provided important SAR information that can be used in future drug designing on this target. The developed QSAR model can also serve as a query model for virtual screening of large libraries. 

### 3.2. Binding Interactions of Protein Residues with Caspase-3 Inhibitors

**Figure 5:**
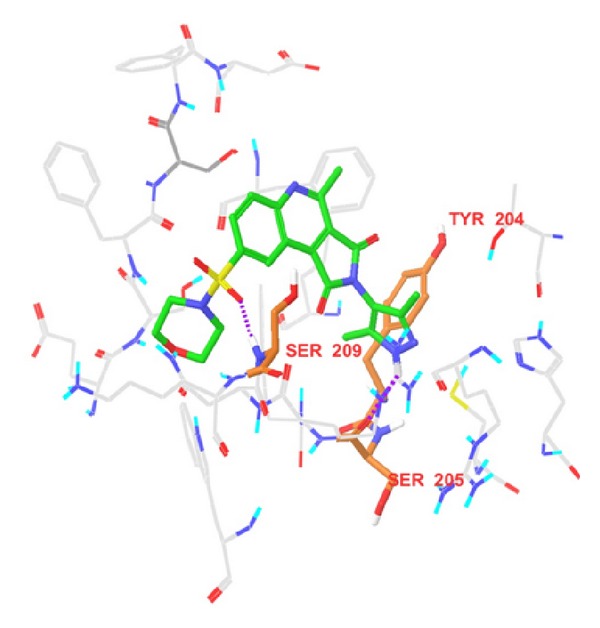
Binding interactions of compound 49 at the active site where the docked ligand is green in color. Hydrogen bonds are expressed as dotted lines in purple color, and active site residues are demonstrated in orange color.

**Figure 6:**
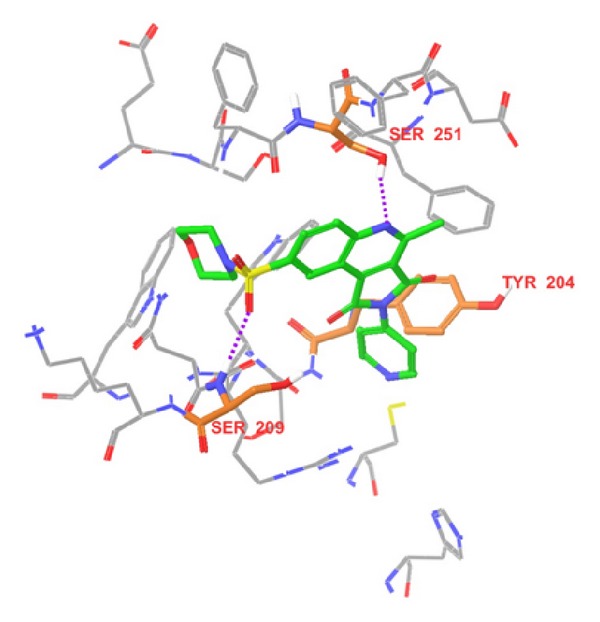
Binding interactions of compound 58 at the active site where the docked ligand is green in color. Hydrogen bonds are expressed as dotted lines in purple color, and active site residues are demonstrated in orange color.

We explored the binding interactions of the two most active compounds (49 and 58) with the receptor. Our aim has been to understand the binding mode of this class of molecules and to cross-check whether the developed pharmacophore model fits properly to the active site. To achieve this, we performed flexible docking using Glide SP mode. Information obtained from binding interactions will be of further help in future design of caspase-3 inhibitors. In addition to analyzing the binding interactions, superposition of most active compounds with respect to common pharmacophore hypothesis was also done. It indicated outstanding superposition of the top two most active compounds. Binding interactions of compound 49 at the active site are depicted in Figure 5 and explained as follows: oxygen atom of SO_2_ group makes hydrogen-bond interaction with NH of Ser 209. NH of aromatic substituent at position 2 makes hydrogen-bond interaction with oxygen atom of Ser 205 and *π*-*π* stacking between aromatic substituent at position 2 and aromatic ring of Tyr 204. Binding interactions of compound 58 at the active site are explained as follows (Figure 6): oxygen atom of SO_2_ group makes hydrogen-bond interaction with NH of Ser 209. Nitrogen atom of quinoline makes hydrogen-bond interaction with OH of Ser 251 and *π*-*π* stacking between aromatic substituent at position 2 and aromatic ring of Tyr 204.

The interactions of both compounds (49 and 58) with surrounding amino acids were also analyzed by MOE molecular modeling software to provide clear view of *π*-*π* stacking between aromatic substituent at position 2 and aromatic ring of Tyr 204. This view is clear from the green line as shown in Figure S_1_ and Figure S_2_ of the Supporting Information. See supplementary Figures S_1_ and S_2_ in Supplementary Material available online at http://dx.doi.org/10.1155/2013/306081.

**Figure 7:**
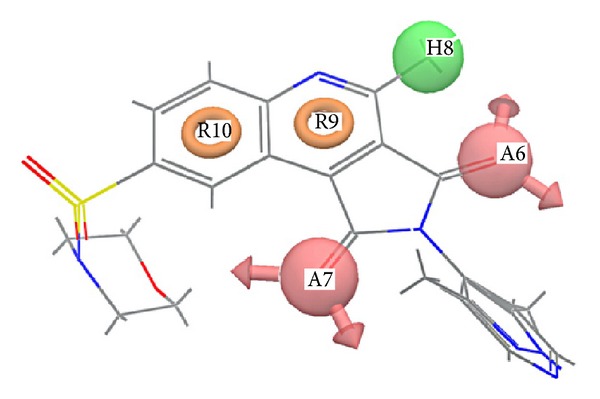
Superposition of compounds 49 and 58 on common pharmacophore hypothesis.

**Figure 8:**
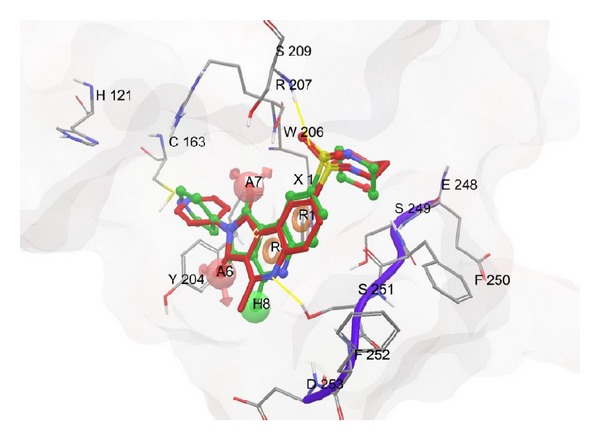
Superposition of pharmacophore hypothesis on docked ligand [compound 58 (X_1_)] at the binding site where the docked ligand is in red color. Hydrogen bonds are expressed in yellow color, and amino acids are expressed in their standard form (H = histidine, C = cysteine, S = serine, R = arginine, W = tryptophan, Y = tyrosine, D = aspartic acid, F = phenylalanine, and E = glutamic acid).

Out of these interactions of the most potent compounds to the active site residues of caspase-3, authors concluded that Ser 209, Ser 251, and Tyr 204 are crucial residues for activity. In addition to these key residues, Ser 205 also has precious contribution to the activity. Since compounds 49 and 58 have similar activities, so they should have almost similar fitness on common pharmacophore, which is clear from Figure 7. Superposition of pharmacophore hypothesis on docked ligand (compound 58) at binding site is depicted in Figure 8.

To cross-check whether the developed pharmacophore model is consistent to the active site residues of caspase-3, we performed the overlay studies. In this study, we aligned the developed pharmacophore on the docked poses of the most potent compound (58) and then manually checked whether the predicted pharmacophoric features were consistent with the protein active site or not. The pharmacophore model predicted the aromatic ring (R) features as an important determinant in biological activity. The docking and the overlay studies revealed a number of favorable aromatic-aromatic and aromatic-aliphatic interactions as shown in Figure S_3_ provided in the Supporting Information [33]. Presence of residues like TRP206, PHE250, and PHE252 and side chains of other aliphatic amino acids around the aromatic feature in the active site is consistent. 

The presence of hydrophobe (H) is also consistent with the docking results, and the presence of side chains of many amino acids like ASP253, PHE256, and SER251 makes favorable hydrophobic interactions. The acceptor feature A7 is also somewhat consistent, due to the presence of ARG207 although we did not observe any hydrogen bonding between the ligand and this residue. The feature A6 predicted by the pharmacophore model is inconsistent and redundant. We also feel that the pharmacophore model may have missed the following important features, which are, otherwise, revealed by the docking studies:

- another aromatic ring feature from the 2nd position of the pyrrolo[3,4-c]quinoline-1,3-diones.
- hydrophobic substituent on sulfonyl group at the 8th position of pyrrolo[3,4-c]quinoline-1,3-diones,

The identification of intrinsic pharmacophoric features strongly depends on structural diversity and composition of the training set compounds. Missing a few probable pharmacophoric features during a ligand-based model building exercise is obvious. Wherever possible, a developed pharmacophore model must be cross-checked for consistency by docking in the protein active site. Missing these features does not undermine the developed pharmacophore model in any way. Instead, it provides an easier and faster way to screen the larger dataset. Therefore, for future virtual screening of caspase-3, we can adopt a strategy of screening the dataset with the developed pharmacophore and then analyze the top HITs by docking or other structure-based studies.

## 4. Conclusion

We were successful in developing an atom-based 3D-QSAR model with high predictive ability. The developed hypothesis consisted of five features with two hydrogen-bond acceptors (A), one hydrophobe (H), and two aromatic rings (R). From the ligand-based study, we can conclude that different electron-withdrawing substituent and with bulkiness at position 8 those heteroaryl substituent at position 2 would be beneficial for designing the new scaffolds of caspase-3 inhibitors. In addition to these suggestions, it is also necessary for a carbonyl group to be electron deficient. These results are also consistent with those of our previous studies. It is further concluded that the developed 3D-QSAR model can also serve as a query model for virtual screening from database to find out new potential caspase-3 inhibitors. Thus, the objective of the present study is to use a computational approach for rapid cost-effective evaluation of caspase-3 inhibitors. These findings can provide direction in order to find out a set of potent caspase-3 inhibitors to be synthesized and examined experimentally for their biological activity. 

## Supplementary Material

The interactions of both compounds (49 and 58) with surrounding amino acids were also analyzed by MOE molecular modeling software to provide clear view of *π*-*π* stacking between aromatic substituent at position 2 and aromatic ring of Tyr 204. This view is clear from the green line as shown in Figure S1 and Figure S2.

## Figures and Tables

**Table 1:** Caspase-3 inhibitors (Series I) with the observed and the predicted biological activities.

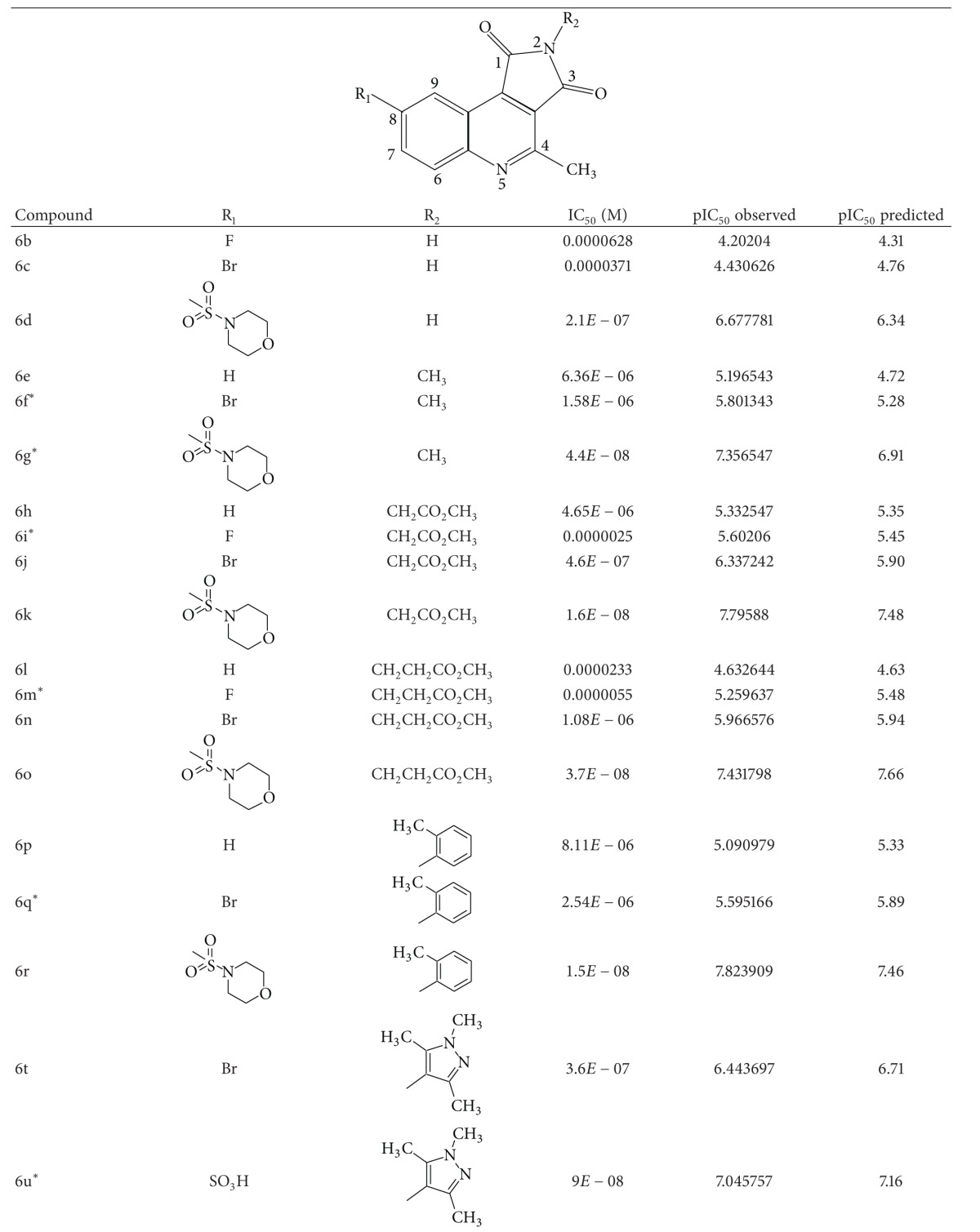 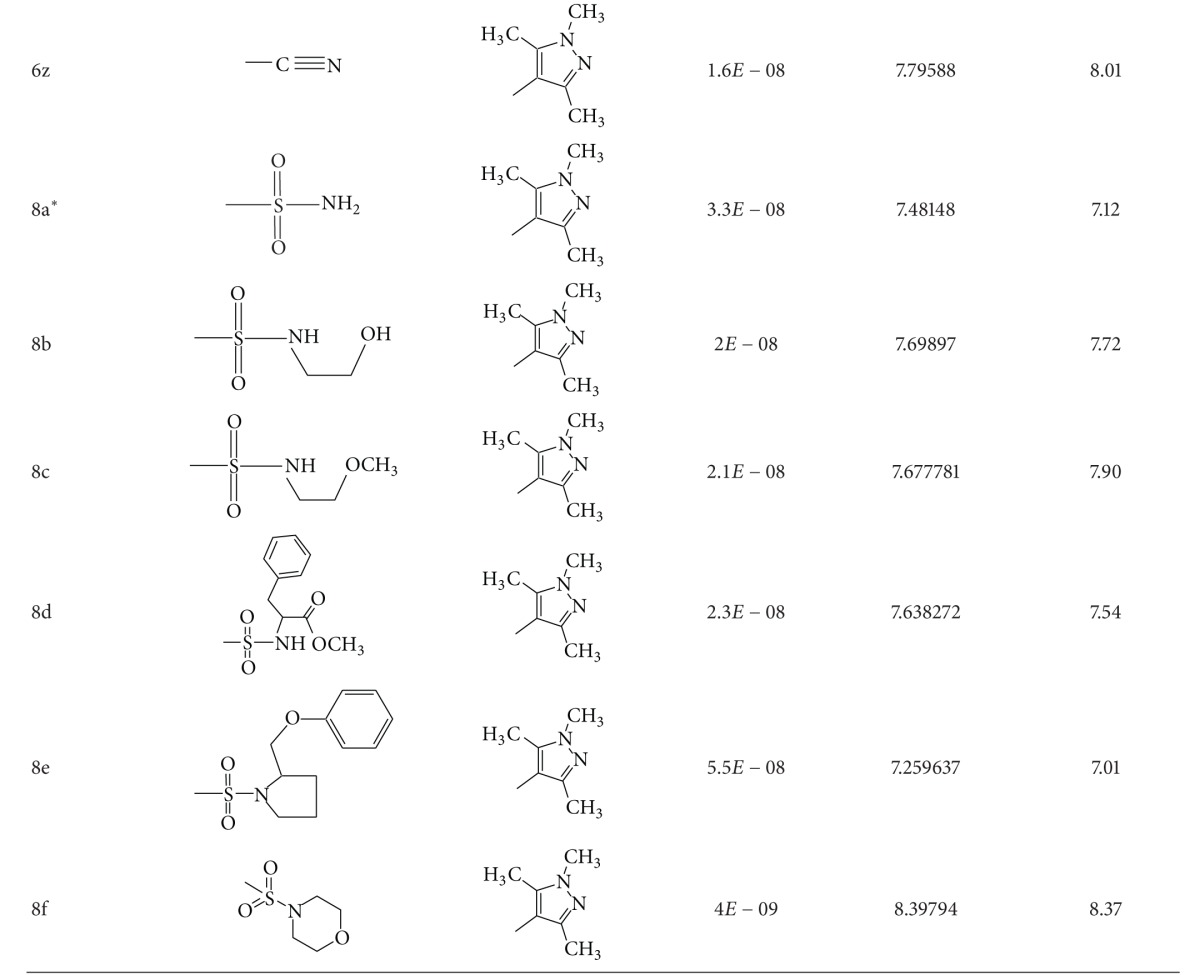

*Compounds involved in the test set.

**Table 2:** Caspase-3 inhibitors (Series II) with the observed and the predicted biological activities.

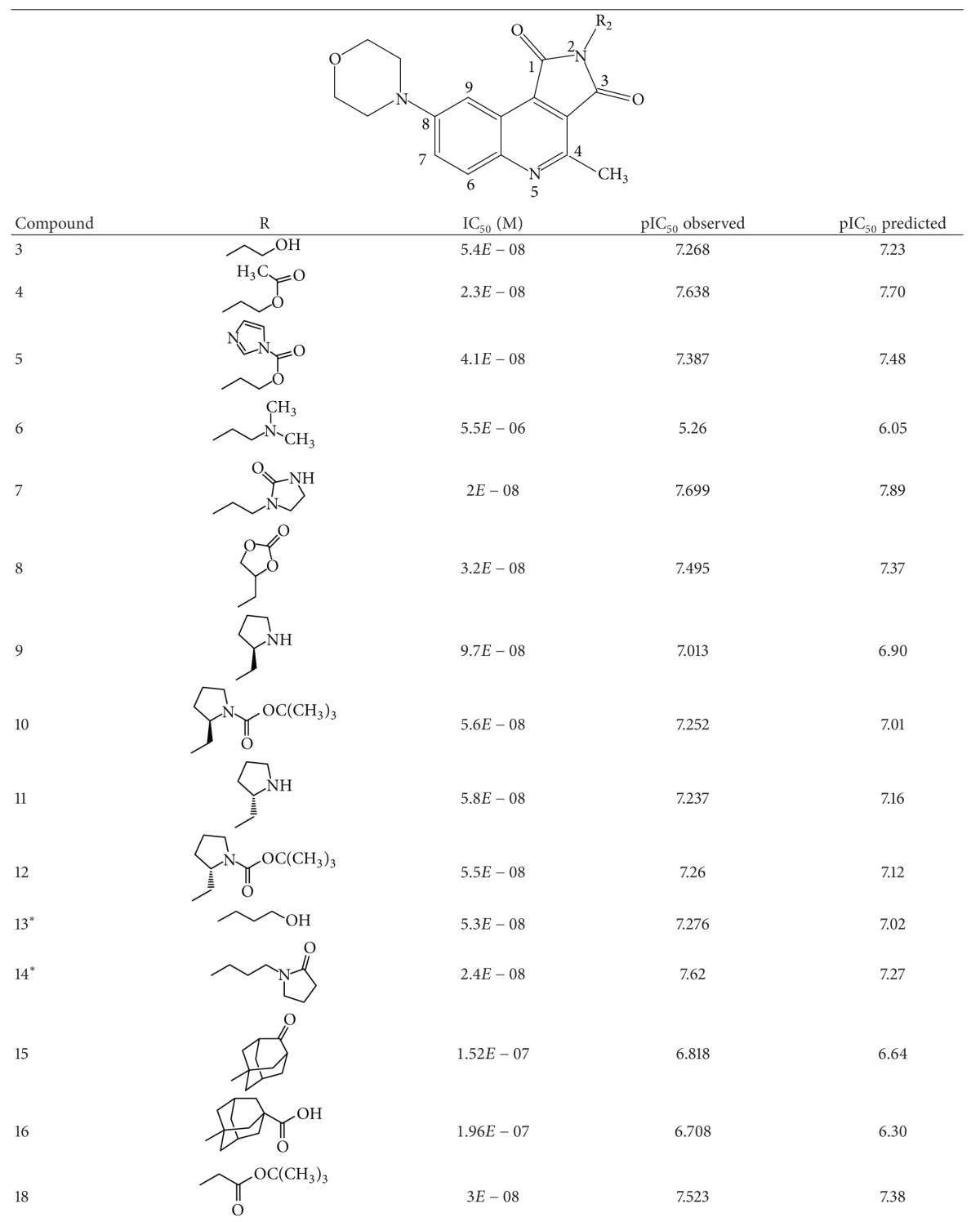 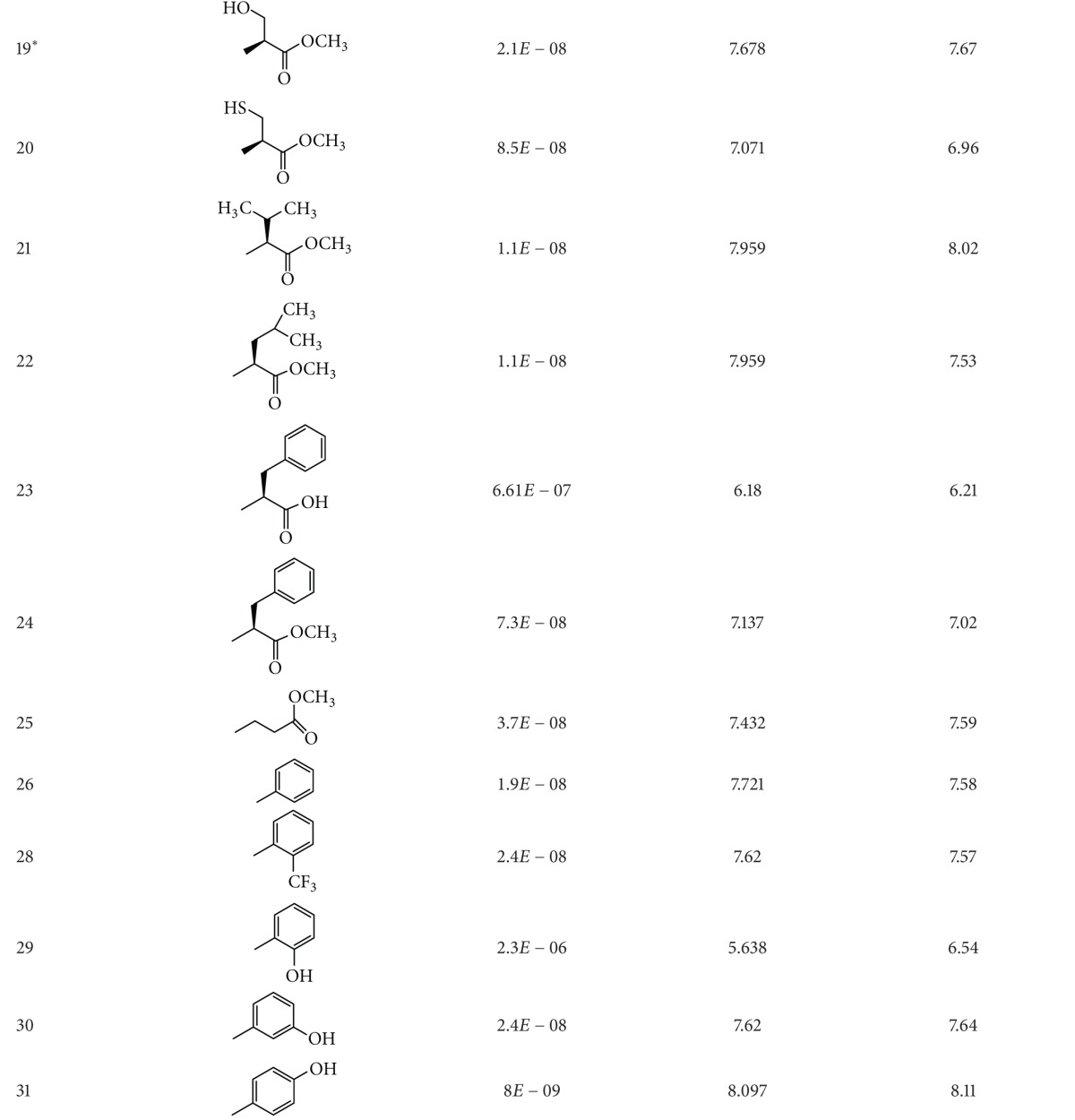 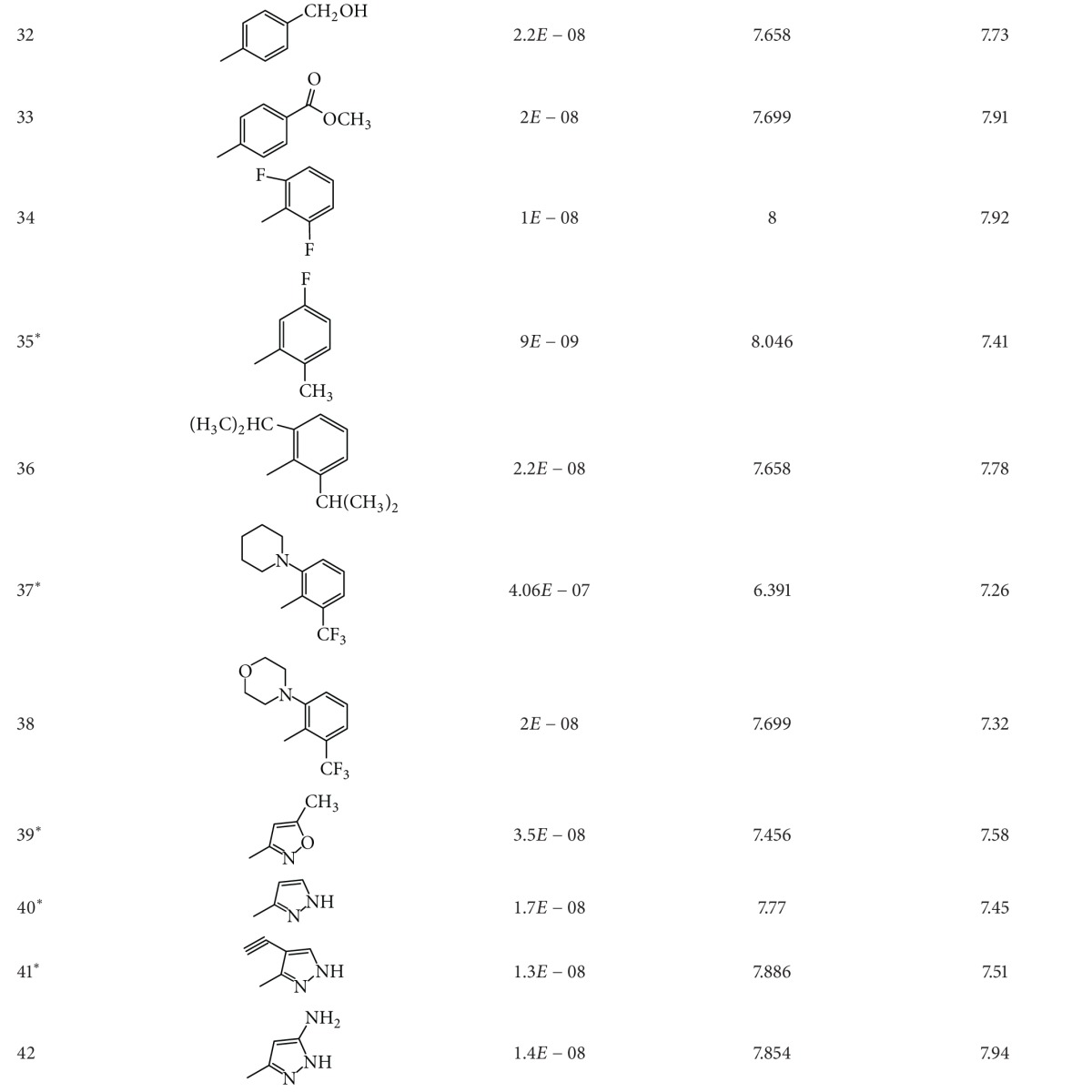 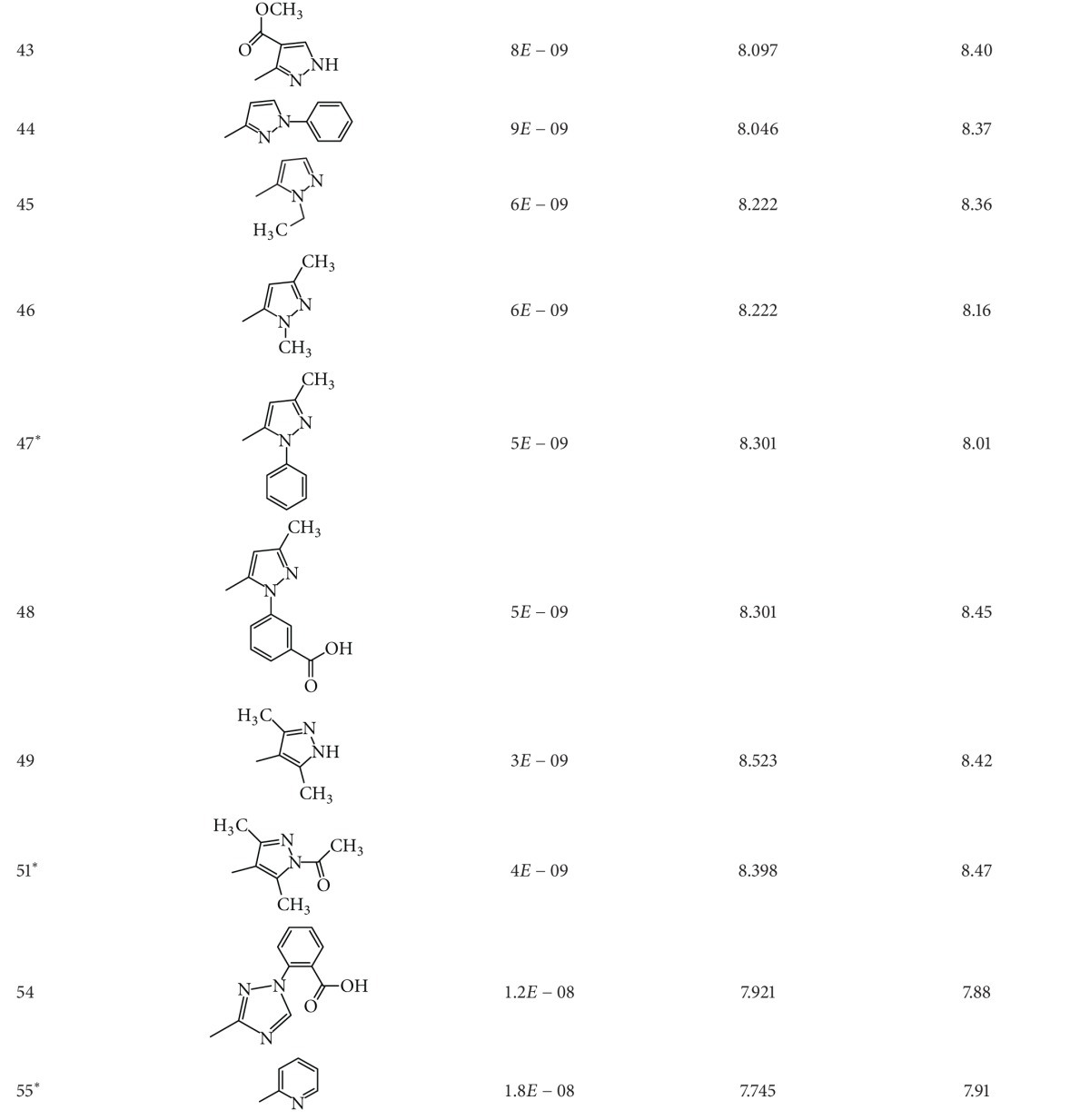 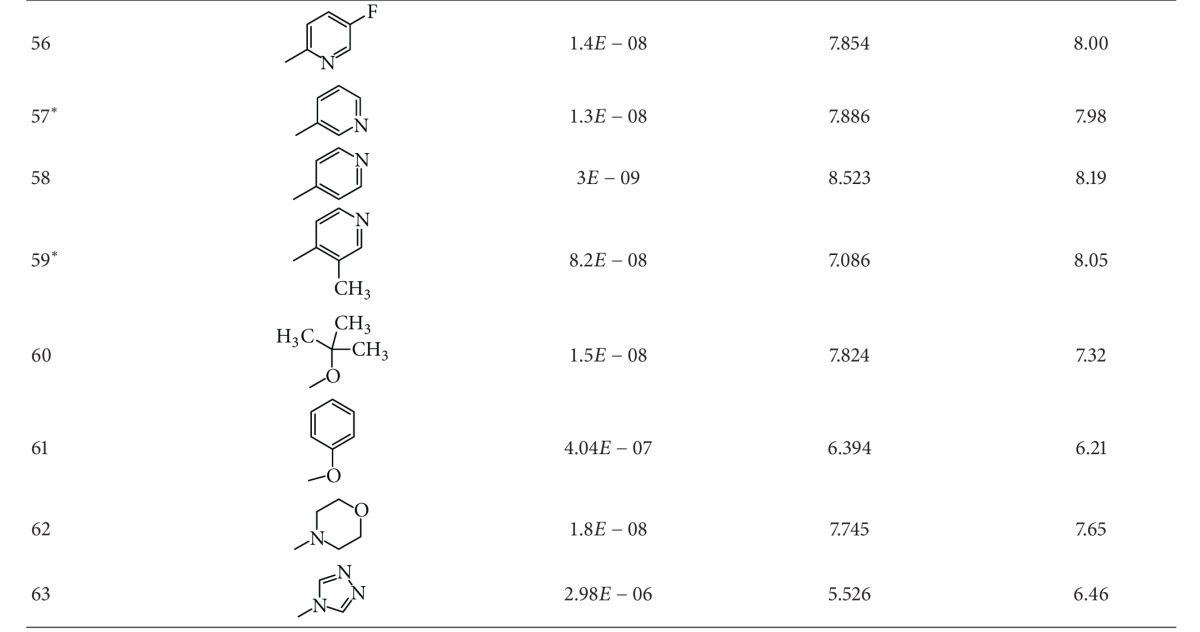

*Compounds involved in the test set.

**Table 3:** Ligand preparation.

Compound	Activity	Conformers	Compound	Activity	Conformers
6b	4.202	1	19	7.678	29
6c	4.431	1	20	7.071	26
6d	6.678	4	21	7.959	9
6e	5.197	1	22	7.959	13
6f	5.801	1	23	6.18	20
6g	7.357	4	24	7.137	12
6h	5.333	2	25	7.432	18
6i	5.602	2	26	7.721	4
6j	6.337	2	28	7.62	5
6k	7.796	8	29	5.638	14
6l	4.633	7	30	7.62	18
6m	5.26	7	31	8.097	8
6n	5.967	7	32	7.658	15
6o	7.432	16	33	7.699	4
6p	5.091	2	34	8	4
6q	5.595	2	35	8.046	6
6r	7.824	7	36	7.658	3
6t	6.444	2	37	6.391	9
6u	7.046	16	38	7.699	8
6z	7.796	2	39	7.456	3
8a	7.481	4	40	7.77	4
8b	7.699	92	41	7.886	8
8c	7.678	35	42	7.854	6
8d	7.638	37	43	8.097	8
8e	7.26	24	44	8.046	7
8f	8.398	8	45	8.222	8
3	7.268	15	46	8.222	7
4	7.638	17	47	8.301	5
5	7.387	32	48	8.301	29
6	5.26	6	49	8.523	8
7	7.699	8	51	8.398	8
8	7.495	17	54	7.921	39
9	7.013	24	55	7.745	5
10	7.252	14	56	7.854	4
11	7.237	27	57	7.886	5
12	7.26	8	58	8.523	4
13	7.276	41	59	7.086	7
14	7.62	35	60	7.824	4
15	6.818	11	61	6.394	6
16	6.708	56	62	7.745	4
18	7.523	16	63	5.526	3

**Table 4:** Score hypothesis.

ID	Survival	Site	Vector	Volume	Selectivity	Matches	Energy	Activity
AAHRR.6	3.785	0.99	1	0.792	1.711	82	0	8.523
AAHRR.7	2.493	0.44	0.595	0.454	1.728	82	0.621	7.62
AAHRR.1	2.463	0.29	0.771	0.4	1.708	82	0.936	7.387
AAHRR.5	2.251	0.19	0.636	0.421	1.71	82	6.812	7.387

**Table 5:** Statistical data of the 3D-QSAR model for PLS factor four.

Training set correlation				Test set correlation		
*R* ^2^	*F* value	SD	*P* value	*Q* ^2^	RMSE	Pearson's *R*
0.925	175.9	0.298	2.336*e* − 31	0.798	0.415	0.897
