# Supplementary material for: Pharmacophore Modeling and Docking Studies on Some Nonpeptide-Based Caspase-3 Inhibitors
Source: Biomed Res Int. 2013 Sep 8;2013:306081. doi: 10.1155/2013/306081 (PMC3780516; doi:10.1155/2013/306081)
Supplement: Supplementary file 1 — The interactions of both compounds (49 and 58) with surrounding amino acids were also analyzed by MOE molecular modeling software to provide clear view of π-π stacking between aromatic substituent at position 2 and aromatic ring of Tyr 204. This view is clear from the green line as shown in Figure S1 and Figure S2. [file 306081.f1.pdf]

Supporting Information

Pharmacophore Modeling and Docking Studies on Some Nonpeptide Based Caspase-3 Inhibitors

Simant Sharma[a], Arijit Basu[b*] and R. K. Agrawal[a*]

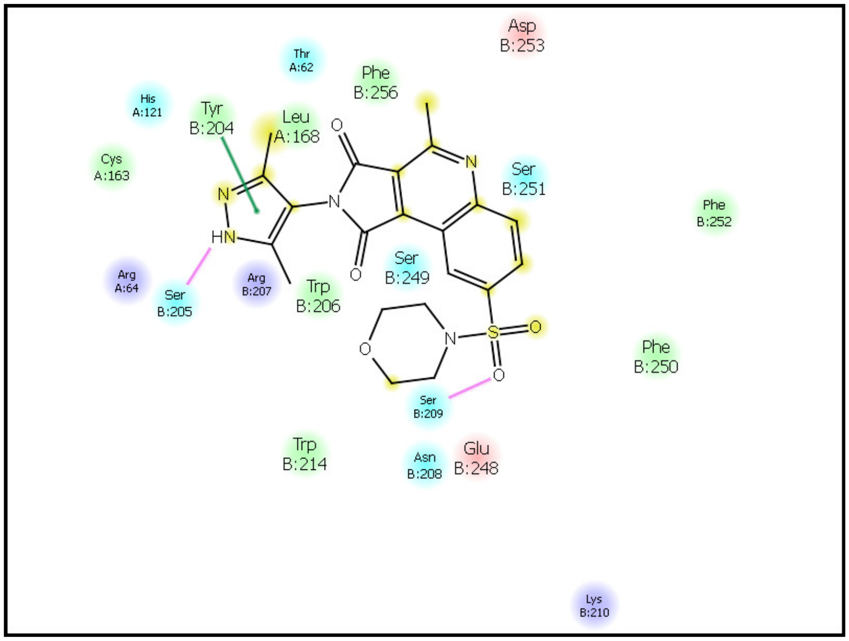

Figure S$_1$: Ligand interaction diagram of compound 49 with receptor where pink sphere: charged (negative); blue sphere: charged (positive); light sky blue sphere: polar residues; light green sphere: hydrophobic; green line is showing π-π stacking between Tyr 204 and aromatic substituent at position 2 of molecule; pink line is showing H-bond (backbone).

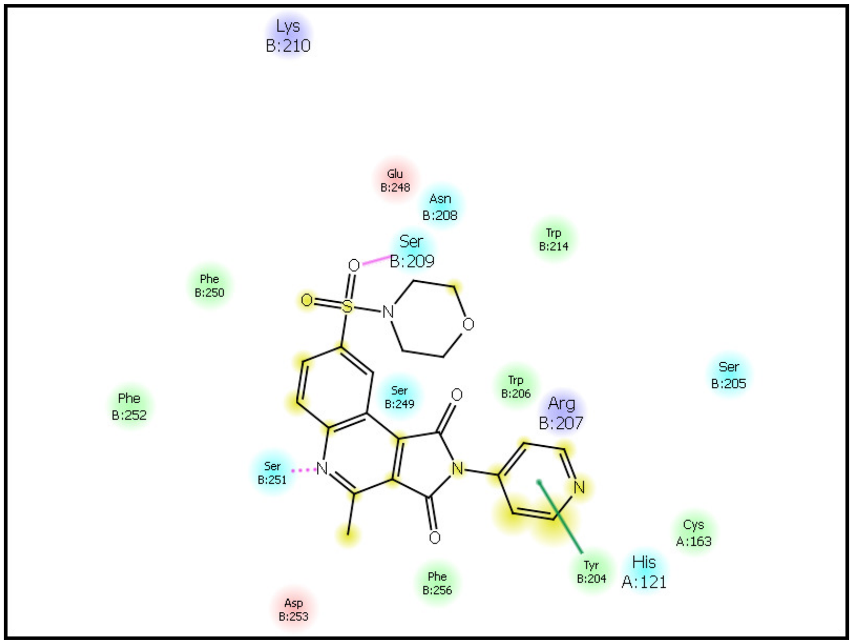

Figure S_2: Ligand interaction diagram of compound 58 with receptor where pink sphere: charged (negative); blue sphere charged (positive); light sky blue sphere: polar residues; light green sphere: hydrophobic; green line is showing π-π stacking between Tyr 204 and aromatic ring of substituent at position 2 of molecule; pink dashed line is showing H-bond (backbone) and pink dotted line is showing H-bond (side chain).

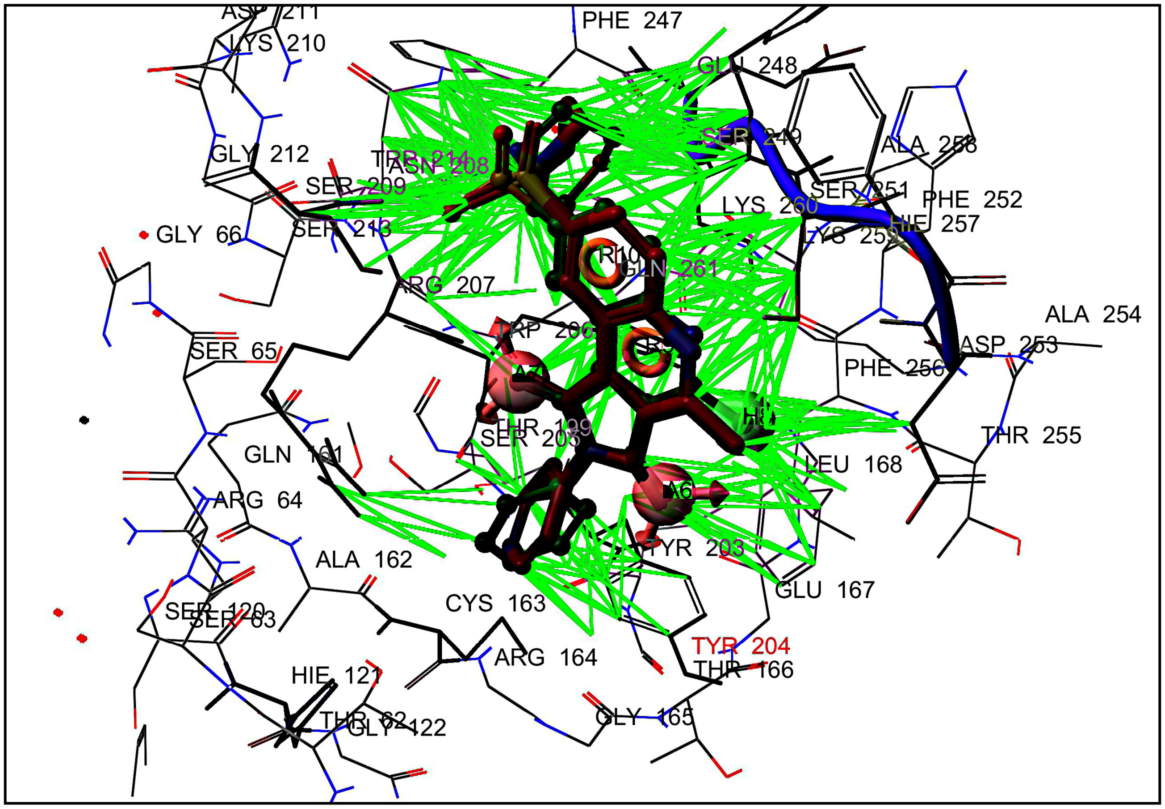

Figure S_3: Overlay study of developed pharmacophore site and docked pose of potent compound (58) and their molecular interactions with active site residues. The figure is showing the consistency of developed pharmacophore features with surrounding residues in binding pocket.
